# The impact of bariatric surgery on macronutrient malabsorption depends on the type of procedure

**DOI:** 10.3389/fnut.2022.1028881

**Published:** 2023-01-12

**Authors:** Charlotte Evenepoel, Greet Vandermeulen, Anja Luypaerts, Daniel Vermeulen, Matthias Lannoo, Bart Van der Schueren, Johan Buyse, Kristin Verbeke

**Affiliations:** ^1^Department of Chronic Diseases, Metabolism and Aging, Translational Research Center for Gastrointestinal Disorders, KU Leuven, Leuven, Belgium; ^2^Laboratory of Lifestock Physiology, Department of Animal and Human Health, KU Leuven, Leuven, Belgium; ^3^Nutrition & Obesity Unit, Clinical and Experimental Endocrinology, Department of Chronic Diseases, Metabolism and Aging, KU Leuven, Leuven, Belgium; ^4^Department of Endocrinology, University Hospitals Leuven, Leuven, Belgium; ^5^Leuven Food Science and Nutrition Research Centre, KU Leuven, Leuven, Belgium

**Keywords:** bariatric surgery, isotope technology, macronutrient malabsorption, protein fermentation, protein digestion

## Abstract

**Introduction:**

Bariatric surgery, currently the most effective treatment for morbidly obese patients, may induce macronutrient malabsorption depending on the type of procedure. Macronutrient malabsorption affects the supply of substrates to the colon, subsequent microbial fermentation and possibly colonic health.

**Methods:**

Using isotope technology, we quantified the extent of macronutrient and bile acid malabsorption and its impact on colonic protein fermentation in patients after Roux-en-Y gastric bypass (RYGB) and sleeve gastrectomy (SG) and in controls. Participants consumed a single test meal (day 0) that contained intrinsically labeled (^13^C, ^15^N, and ^2^H) egg protein for quantification of protein digestion, malabsorption and fermentation, respectively, together with a transit marker and a marker for bile acid malabsorption. They collected breath samples up to 6 h and all urine and stool for 48 and 72 h, respectively. Food intake was registered from day –3 to day 2.

**Results:**

Malabsorption of fat, protein and carbohydrates differed between groups (*p* = 0.040; *p* = 0.046; and *p* = 0.003, respectively) and was slightly higher in RYGB but not in SG patients compared to controls. Protein fermentation was increased in both RYGB and SG patients compared to controls (*p* = 0.001) and was negatively correlated to ^2^H-recovery as a marker of transit (ρ = −0.47, *p* = 0.013).

**Conclusion:**

The limited macronutrient malabsorption likely does not affect the nutritional status of the patient. However, the higher protein fermentation may affect colonic health and warrants further investigation.

## Introduction

Bariatric surgery (BS) is currently the most efficient treatment to induce long-term weight loss and improve health in patients with morbid obesity (1). Worldwide, laparoscopic Roux-en-Y gastric bypass (RYGB) and sleeve gastrectomy (SG) are the most common types of BS (2). After RYGB, the stomach is converted to a small gastric pouch and a large part of the small intestine is bypassed. SG only involves a vertical resection of the stomach along the greater curvature, leaving the intestines intact (3).

Originally, these procedures were intended to induce weight loss by restricting caloric intake (small gastric volume) and, in the case of RYGB, by reducing nutrient absorption in the small intestine (reduced absorptive surface) (4). Nowadays, several studies have associated the increased secretion of the gut hormones GLP-1, CCK, and PYY, altered bile acid signaling and microbiota composition to the weight loss and the metabolic benefits seen after RYBG and SG (5, 6).

Even if malabsorption is not a major driver for weight loss after BS, it remains essential to quantify the extent of macronutrient malabsorption for several reasons. First, knowledge on the extent of macronutrient malabsorption may improve dietary guidelines for patients after surgery and aid in preventing undernutrition (7, 8). Secondly, food components that are not digested and absorbed in the small intestine, reach the colon where they serve as substrate for the residing microbiota. Changes in substrate availability may drive the alterations in microbiota composition observed after BS resulting in altered interactions with bile acids and farnesoid X receptor (FXR) signaling. Activation of the FXR regulates hepatic bile acid metabolism, glucose and lipid metabolism (9). Thirdly, changes in supply of substrates to the colonic microbiota also affect the type and amount of metabolites produced by bacterial fermentation. Those metabolites are in close contact with the intestinal cells and play a role in colonic health. Short chain fatty acids are the main metabolites from carbohydrate fermentation and are considered beneficial. In contrast, some protein fermentation metabolites such as p-cresol, ammonia and hydrogen sulfide may harm colonic cells by disrupting the epithelial barrier and inducing DNA damage (10, 11).

In this study, we applied isotope technology to quantify macronutrient and bile acid malabsorption in weight-stable RYGB and SG patients (6–18 months after surgery), and in a control group. Furthermore, we evaluated the impact of surgery on colonic protein fermentation. As the small intestinal anatomy is rearranged after RYGB but not SG, we hypothesized that macronutrient malabsorption is affected to a greater extent after RYGB than after SG.

## Materials and methods

### Subjects

Roux-en-Y gastric bypass and SG patients that had surgery 6–18 months ago were recruited at the Obesity Clinic of the University Hospital Leuven (Leuven, Belgium). Unoperated subjects with a BMI between 18 and 30 kg/m^2^ were recruited as controls (CTR). Exclusion criteria were abdominal surgery (except appendectomy, cholecystectomy, and bariatric surgery) and kidney, liver, lung and gastro-intestinal disease. Participants were free from antibiotics and pre-and probiotics for 1 month and from antidiarrheal drugs and laxatives for 2 weeks prior to the start of the study. Subjects on a specific diet, including a vegan, vegetarian, lactose-or gluten-free diet, pregnant or breastfeeding women and subjects that participated in a clinical study with radiation exposure in the past year were excluded. The study was approved by the Ethics Committee of UZ/KU Leuven and registered at ClinicalTrials.Gov (clinical trial number: NCT04345328). All subjects signed written informed consent.

### Study design

**FIGURE 1:**
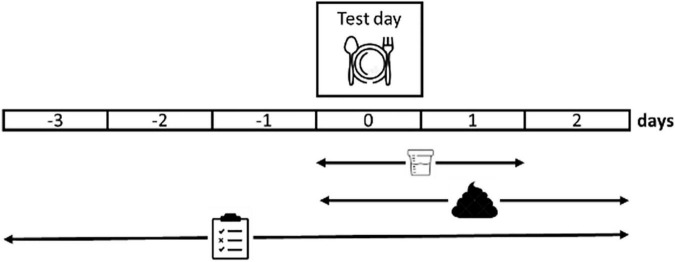
Schematic overview of the study design. Participants self-report their dietary intake from 3 days before a test day (day 0) up to 2 days after the test day. The test day is performed in the laboratory and consists of the consumption of an isotope labeled standard meal and the collection of breath samples (6 h), urine and feces. Urine collection is continued up to 48 h and feces collection up to 72 h after the standard meal.

Participants performed a single test day (day 0) but started to complete a dietary record at home at day −3. The study is schematically presented in Figure 1 and the details of all procedures are described in the subsequent paragraphs. On day −1, they were asked to avoid fiber-rich food (only white bread, white pasta, and white rice, maximal one piece of fruit and no cabbage, legumes, or onions) and alcohol. On the morning of day 0, they came to the laboratory in fasted state for the test day. After collection of baseline breath samples, participants consumed an intrinsically stable isotope labeled pancake test meal with a glass of water, a gelatine capsule containing ^14^C-glycocholic acid (185 kBq, PerkinElmer, Shelton, CT, USA) to assess bile acid malabsorption and a capsule with ^3^H-polyethylene glycol (^3^H-PEG, 185 kBq, PerkinElmer) as a marker of total transit. Additional breath samples for analysis of ^13^C, ^14^C, and H_2_ were collected at regular time points up to 6 h after the test meal. Upon completion of the breath sampling, participants left the laboratory and could have lunch and dinner *ad libitum* but kept recording food intake. Furthermore, they collected all stools for the next 72 h and stored them immediately at −20°C. Urine was collected for 48 h in dedicated recipients to which 500 mg neomycin was added to prevent bacterial growth. On day 3, all samples and diaries were returned to the lab. The fecal collection was weighed, homogenized and lyophilized. Urine samples were weighed, aliquoted and stored at −20°C until analysis.

### Production of stable isotope labeled eggs

Eggs were intrinsically labeled with ^13^C, ^15^N, and ^2^H by feeding 2 laying hens (Hisex white, body weight of about 2 kg) at peak egg production with standard feed (Farm 3 Mash, HobbyFirst, Schoten, Belgium) supplemented (3 g/kg) with L-[1-^13^C]-leucine, L-[^15^N]-leucine and L-[ring-^2^H_5_]-phenylalanine (Euriso-top, Saint-Aubin, France; >98 mole%). The hens incorporated these amino acids into their egg proteins. Dietary L-[ring-^2^H_5_]-phenylalanine is partly hydroxylated by the hen’s metabolism and incorporated into the egg protein as both L-[ring-^2^H_4_]-tyrosine and L-[ring-^2^H_5_]-phenylalanine. From day 14, the eggs were collected daily and lyophilized. The ^15^N- and ^13^C-abundance was measured using an elemental analyzer coupled to an isotope ratio mass spectrometry (IRMS) (ANCA-2020, Europe Scientific, Crew, UK). The ^2^H-abundance of the eggs was assessed with gas chromatography mass spectrometry (GC-MS, Trace GC 1300 and DSQ II XL, Thermo Electron Corporation, Wathham, MA, USA), after hydrolysis of the egg proteins and derivatization of the resulting amino acids, as described previously (12). The pooled eggs had a ^13^C-abundance of 1.18 atom%, a ^15^N-abundance of 1.64 atom% and a L-[ring-^2^H_4_]-tyrosine abundance of 3.78 mole%.

### Pancake test meal

The pancake test meal was prepared by adding 100 mL of water to a mix of 24.7 g of lyophilized labeled egg, 3.75 g of unlabeled lyophilized egg white, 17 g of wheat flour, 7 g of sugar, 3 g of milk powder and baking the resulting dough with 6 g of butter. The pancake was consumed with an additional 5 g of sugar. The total caloric content of the meal was 326 kcal and consisted of 18 g of protein, 16 g of fat and 27 g of carbohydrates (13).

### Self-reported dietary intake

Participants digitally registered their food intake for 6 days using the mobile application or website of MyFitnessPal (MyFP). To ensure accurate registration, we provided a manual on how to use MyFP. Participants were asked to weigh all consumed food and drinks on a kitchen scale. Upon delivery, the food diaries were checked for irregularities and corrected if required. Macronutrient composition was calculated manually using the portion size and the nutritional information from the label of branded items and from the Belgian Composition Data Table for generic items. Results were expressed in absolute amounts (g/day) or as% of energy intake (EN%) using 4 kcal/g for protein and carbohydrate, 9 kcal/g for fat and 2 kcal/g for dietary fiber.

### Protein digestion

Protein digestion was estimated from the rate of ^13^CO_2_ appearance in breath after ingestion of intrinsically labeled proteins (14, 15). Breath samples for quantification of ^13^C were collected by blowing through a straw in an Exetainer^®^ (Labco Ltd., Ceredigion, UK) and the isotopic abundance of CO_2_ was measured using IRMS (ABCA, Sercon, Crewe, UK). CO_2_-production was assumed to be 300 mmol per m^2^ body surface area per hour, with body surface area being calculated by the weight-height formula of Haycock et al. (16). Results were expressed as% of administered dose of ^13^C recovered per hour (^13^C-recovery/h) and as cumulative% of administered dose of ^13^C over 6 h (6 h ^13^C-recovery).

### Bile acid malabsorption

A ^14^C-glycocholic acid breath test was used to assess bile acid malabsorption (17). Breath samples for analysis of ^14^C were collected by blowing through a pipette into a vial containing 2 mmol of hyamine hydroxide until discoloration of the thymolphtalein color indicator, which corresponds to the capture of 2 mmol CO_2_. The amount of ^14^CO_2_ was measured by β-scintillation counting (PerkinElmer, Shelton, CT, USA) after addition of 10 ml Emulsifier-Safe™ (PerkinElmer). Results were expressed as disintegrations per minute (dpm) and converted to% of administered dose of ^14^C/h (^14^C-recovery/h). Results were classified as positive or negative for bile acid malabsorption based on visual inspection of the curves.

### Carbohydrate malabsorption

As hydrogen is not produced by mammalian enzymes but only upon bacterial metabolism of carbohydrates, excretion of hydrogen (H_2_) in breath was measured as an indication of malabsorption of the carbohydrates (starch and sugar) in the pancake test meal (18). Breath H_2_ was measured using GC with thermal conductivity detection (19) and used to calculate the positive incremental area under the curve (iAUC).

### Fat malabsorption

Fat malabsorption was expressed as% fat excretion ([fat excretion/fat intake] × 100). Fat intake was calculated from the food diary analysis and was expressed as g fat/day. Excretion of fat in feces was quantified by extracting 200 mg lyophilized feces in petroleum ether, using a continuous Soxhlet extraction (20). The petroleum ether extract was dried and the amount of fat measured gravimetrically. Results were expressed as g fat excreted per day.

### ^3^H-recovery

The tritium (^3^H) content in lyophilized fecal samples was measured by liquid scintillation counting after oxidation to [^3^H]-H_2_0 (Sample Oxidizer, model 307, PerkinElmer). Results of ^3^H-recovery were expressed as% of the administered ^3^H dose recovered over 72 h. This 72-h ^3^H-recovery was used as a measure of total transit (21).

### Protein malabsorption

Protein malabsorption was calculated as the% difference between ^15^N intake and fecal ^15^N excretion. Intake of ^15^N was calculated from the measured enrichment and total N content of the labeled egg. For ^15^N excretion, total N content and ^15^N-abundance of lyophilized fecal samples were determined (22). Total amount of excreted ^15^N was calculated from the amount of ^15^N in the fecal sample, the% of dry weight and the total fecal output. The ^15^N-recovery in feces over 72 h was corrected for total transit by dividing the% ^15^N recovery by the% ^3^H recovery.

### Protein fermentation

Urinary recovery of p-[ring-^2^H_4_]cresol was measured to estimate protein fermentation. After thawing, urine was analyzed for p-[ring-^2^H_4_]-cresol content using GC-MS, as described previously (23). Results were expressed as the% of administered dose of L-ring[^2^H_4_]tyrosine.

### Statistical analysis

Sample size calculation was performed using protein malabsorption as the outcome because we considered protein malabsorption as an important factor impacting colonic fermentation and gut health. Based on previous data from our lab that indicate a 5.7% protein malabsorption in physiological conditions (24) and an increase of 20% protein malabsorption reported after RYGB (25), we calculated that 10 subjects in each group would be sufficient to provide a 90% chance for detecting such difference at the 5% level of significance. Unfortunately, we managed to only include 8 SG patients that fulfilled all criteria.

The residuals of all variables were tested for normality and equal variance using a Shapiro-Wilk and Levene’s test, respectively. In case normality or equal variance were not obtained after transformation, Kruskall–Wallis or Welch’s ANOVA, respectively, was applied. For *post-hoc* pairwise comparisons, the Tukey–Kramer test was used. Continuous variables were compared across the 3 groups, using a one-way ANOVA. Whereas a Fisher’s Exact test was applied for categorical variables. Using ANCOVA, group comparison of protein fermentation was adjusted for 72-h ^3^H-recovery, protein malabsorption and fiber intake, whereas ^3^H-recovery was adjusted for fiber intake. Fat malabsorption was adjusted for bile acid malabsorption status. Additionally, Pearson or Spearman correlations were applied, depending on the normality of the data. Data were analyzed using SAS 9.4 (SAS Institute, Cary, NC, USA). Significance level was set at a *p*-value < 0.05.

## Results

### Study population

Ten RYGB patients, 8 SG patients, and 10 CTR were included in the study. From the 113 subjects screened, 85 did not meet the inclusion criteria or did not wish to participate. One SG patient had insufficient CO_2_ in the breath samples for reliable ^13^C-abundance analysis and was excluded from the protein digestion analysis.

Age, weight, and BMI did not differ across the three groups (Table 1). Time after surgery was similar for both surgery groups and the proportion of men and women did not differ across the groups.

**TABLE 1:** Demographic characteristics of the participants.

	CTR (*N* = 10)	RYGB (*N* = 10)	SG (*N* = 8)	*p*-Value
Age (years)	44.60(12.45)	44.00(11.71)	36.63(14.68)	0.374
Weight (kg)	77.48(14.56)	78.34(16.40)	91.74(16.20)	0.116
BMI (kg/m^2^)	25.81(2.02)	26.91(3.34)	29.16(3.06)	0.061
Time after surgery (months)	NA	8.90(2.47)	10.50(4.50)	0.414
Gender (M/F)	4/6	2/8	5/3	0.248

Data are expressed as mean(SD). One-way ANOVA was performed to compare group means. A Fisher’s Exact test was performed to compare the distribution of categorical data. CTR, control group; RYGB, Roux-en-Y Gastric Bypass; SG = Sleeve Gastrectomy.

### Dietary intake

Total energy intake was lower in the surgery groups than in the CTR group (Table 2). RYGB patients ate significantly less carbohydrates and fat but similar amounts of protein compared to CTR, resulting in a higher EN% from protein in the RYGB than in the CTR group (*p* = 0.029). EN% from carbohydrates and fats was not different between groups. Fiber intake in SG but not RYGB patients was lower than in CTR (*p* = 0.033 and *p* = 0.622, respectively).

**TABLE 2:** Self-reported total energy, macronutrient and fiber intake

	CTR (N=10)	RYGB (N=10)	SG (N=8)	p-value*
**Total energy**				
kcal/day	1955(363) ^a^	1427(287) ^b^	1546(332) ^b^	0.004
**Carbohydrates**				
g/day	213(48) ^a^	152(41) ^b^	170(48) ^a^	0.016
EN%	43(4)	42(7)	44(6)	0.865
**Fibre**				
g/day	15(6) ^a^	12(5) ^a^	8(3) ^b^	0.040
EN%	2(1)	2(1)	1(0)	0.078
**Fat**				
g/day	77(17) ^a^	56(12) ^b^	63(19) ^a^	0.023
EN%	36(4)	36(5)	36(5)	0.908
**Protein**				
g/day	81(13)	76(21)	72(24)	0.613
EN%	17(2) ^a^	21(4) ^b^	19(6) ^a^	0.034

Values are expressed as mean(SD).*The p-value refers to the significance of the one-way ANOVA. Superscript Letters in superscript (a,b) refer to the pairwise comparisons between groups. Values with different letters indicate significant differences (p<0.05). EN% = energy percentage, CTR = control, RYGB = Roux-en-Y Gastric Bypass and SG = Sleeve Gastrectomy.

### ^3^H-recovery

**FIGURE 2:**
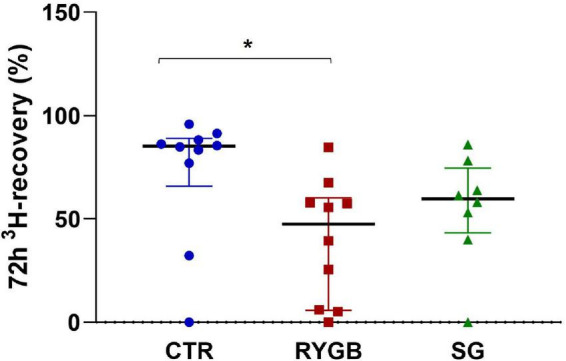
Median (IQR) 72-h ^3^H-recovery in feces of controls and bariatric surgery patients. Asterix indicates significance at the 0.05 level (one-way ANOVA with *post-hoc* Tukey–Kramer). CTR, control; RYGB, Roux-and-Y Gastric Bypass; SG, sleeve gastrectomy.

^3^H-recovery was different between groups (ANOVA, *p* = 0.005). RYGB patients had a lower ^3^H-recovery than CTR, reflecting a slower transit (*p* = 0.005, Figure 2), whereas SG did not differ from RYGB (*p* = 0.665) and had a borderline non-significant difference with CTR (*p* = 0.054). Surprisingly, fiber intake did not correlate with total transit (ρ = 0.19, *p* = 0.325). After correction for fiber intake, the group effect on 72-h ^3^H-recovery remained significant (*p* = 0.012).

### Protein digestion, malabsorption, and fermentation

**FIGURE 3:**
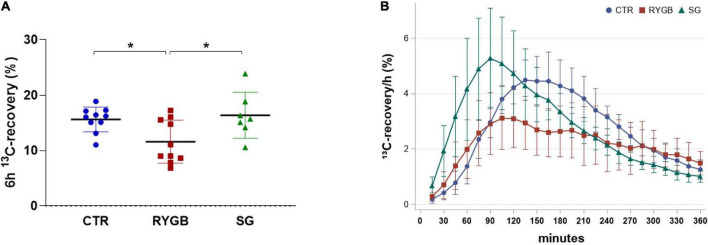
^13^C-recovery in breath of controls and bariatric surgery patients. **(A)** Mean (SD) 6-h ^13^C-recovery and **(B)** ^13^C-recovery/h over time. Values are displayed as mean (SD) and *N* = 27. Asterix indicates significance at the 0.05 level (one-way ANOVA with *post-hoc* Tukey–Kramer). CTR, control; RYGB, Roux-and-Y Gastric Bypass; SG, sleeve gastrectomy.

Breath 6-h ^13^C-recovery was different across groups (ANOVA *p* = 0.015), with lower recovery in RYGB patients than in CTR (*p* = 0.039) and SG patients (*p* = 0.025; Figure 3A), indicating a lower protein digestion after RYGB. SG patients did not differ from CTR (*p* = 0.901). Time to maximum ^13^C-recovery (t_max_) differed across the groups (ANOVA *p* = 0.003) with the surgery groups having a shorter t_max_ (110 and 105 min, for RYGB and SG patients, respectively) than the CTR group (150 min) (Figure 3B).

**FIGURE 4:**
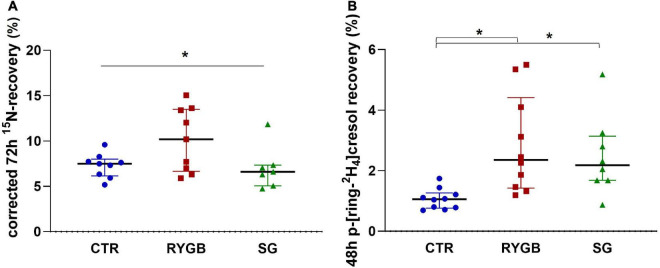
**(A)** Median (IQR) 72-h ^15^N-recovery (% ^15^N × 100/% ^3^H) in feces and **(B)** median (IQR) 48-h p-ring[^2^H_4_]cresol recovery (% of administered *p*-ring[^2^H_4_]tyrosine) in urine of controls and bariatric surgery patients. Asterix indicates significance at the 0.05 level (one-way ANOVA with *post-hoc* Tukey–Kramer). CTR, control; RYGB, Roux-and-Y Gastric Bypass; SG, sleeve gastrectomy.

The corrected 72-h ^15^N-recovery differed between the three groups (ANOVA *p* = 0.046; Figure 4A) although pairwise comparisons did not yield differences between groups. After exclusion of the outlier in the SG group, ^15^N-recovery was higher after RYGB than after SG (*p* = 0.016). Higher protein malabsorption was associated with lower protein digestion (ρ = −0.47, *p* = 0.020).

Protein fermentation, evaluated from the p-[ring-^2^H_4_]cresol-recovery in urine, was different across groups (ANOVA *p* = 0.001), with higher fermentation in both surgery groups than in the CTR group (RYGB: *p* = 0.001 and SG: *p* = 0.006; Figure 4B) but no difference between surgery groups (*p* = 0.856). The p-[ring-^2^H_4_]cresol-recovery correlated negatively with ^3^H-recovery (ρ = −0.47, *p* = 0.013), indicating that a higher degree of protein fermentation was associated with a slower total transit. In contrast, protein fermentation did not correlate with protein malabsorption (ρ = 0.13, *p* = 0.524), nor with fiber intake (ρ = −0.19, *p* = 0.334). Group had an effect on protein fermentation, regardless of ^3^H-recovery (*p* = 0.003), protein malabsorption (*p* = 0.005) or fiber intake (*p* = 0.002).

### Carbohydrate malabsorption

**FIGURE 5:**
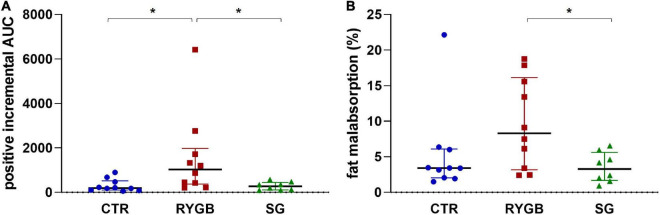
**(A)** Median (IQR) positive iAUC of H_2_ in breath and **(B)** median (IQR) fat malabsorption. Asterix indicates significance at the 0.05 level (one-way ANOVA with *post-hoc* Tukey–Kramer). AUC, area under the curve; RYGB, Roux-en-Y gastric bypass; SG, sleeve gastrectomy; CTR, control.

The iAUC of breath H_2_ was different across group (ANOVA *p* = 0.003). The RYGB group had higher breath H_2_ excretion than CTR and SG, (Figure 5A, *p* = 0.005 and *p* = 0.014, respectively), indicating more carbohydrate malabsorption after RYGB. CTR and SG subjects exhibited a similar extent of carbohydrate malabsorption (*p* = 0.978, Figure 5A).

### Fat malabsorption

Fat malabsorption differed across group (ANOVA *p* = 0.040) and was higher in RYGB patients than in SG patients (*p* = 0.043; Figure 5B). The CTR group did not differ from the surgery groups (RYGB: *p* = 0.138 and SG: *p* = 0.766) due to the presence of an outlier. After exclusion of this outlier, fat malabsorption was higher in RYGB than CTR (*p* = 0.025). The group effect on fat malabsorption remained significant (*p* = 0.004) after adjusting for bile acid malabsorption status.

### Bile acid malabsorption

BS did not influence bile acid malabsorption as the proportion of subjects with bile acid malabsorption was similar across the three groups (*p* = 1.00). Only 1 CTR and 1 RYGB patient suffered from bile acid malabsorption.

## Discussion

We hypothesized that the impact of BS on the assimilation of nutrients depends on the type of procedure. As the extent of anatomical rearrangement and nutrient rerouting is more pronounced after RYGB than after SG, we expected a higher level of malabsorption after RYGB. Indeed, our results indicate that RYGB, but not SG, slightly increased fat, protein and carbohydrate malabsorption compared to a control group with similar BMI that did not undergo surgery. Remarkably, the extent of protein fermentation was not only higher in RYGB but also in SG patients.

Fat malabsorption after RYGB has been reported previously (25–27) and is confirmed in the present study. In contrast, SG does not induce fat malabsorption. Absorption of dietary fat requires the concerted action of pancreatic lipase, to hydrolyze the triglycerides in the small intestine, and bile acids to form micelles for absorption of the fatty acids. A disturbance of either process will result in fat malabsorption. Occurrence of bile acid malabsorption could be ruled out as a causative factor since the bile acid breath test showed similar results across the groups. Similarly, previous studies comparing fecal bile acid secretion before and after RYGB excluded bile acid malabsorption (25, 26). Secretion of pancreatic enzymes is induced by secretion of cholecystokinin and secretin upon exposure of duodenal enteric endocrine cells to nutrients (28). As the duodenum is bypassed after RYGB, pancreatic stimulation is reduced resulting in lower secretion of digestive enzymes (29). Also asynchronization between the release of enzymes and passage of nutrients as a result of the structural changes to the GI tract may reduce fat digestion (30). Furthermore, supplementation of RYGB patients with pancreatic supplements (40,000 USP units per meal for 3 months) reduced, but did not normalize, the fat malabsorption, indicating that the malabsorption was at least partly due to enzyme deficiency (30). The fact that the duodenum remains intact after SG may explain the preservation of normal fat digestion after SG. As luminal digestion of fat is hardly compensated by non-pancreatic mechanisms, malabsorption of fat and steatorrhea are generally more severe and occur before malabsorption of protein and carbohydrates in case of pancreatic insufficiency (31). Hence, it is not surprising that malabsorption of fat is the most pronounced finding after BS.

We used egg proteins intrinsically labeled with three stable isotopes (^13^C, ^15^N, and ^2^H) to non-invasively evaluate the extent of protein assimilation, i.e., digestion, malabsorption, and fermentation. The ^13^C-protein breath test is a validated tool to quantify the extent of small intestinal protein digestion (32). The fractional fecal loss of ^15^N from orally administered egg protein was quantified as a measure of protein malabsorption. Quantified in this way, protein malabsorption might be overestimated due to tracer recycling, i.e., secretion into the colon of ^15^N that had been absorbed in the small intestine (33). However, previous studies in our lab using this technique showed that protein malabsorption was slightly lower in healthy subjects compared to healthy ileostomy subjects in identical conditions (24), suggesting a slight underestimation rather than overestimation of the protein losses, probably due to nitrogen salvage from the colon (34). Finally, the extent of protein fermentation was estimated from the urinary recovery of p-[ring-^2^H_4_]cresol (23). p-[ring-^2^H_4_]Cresol is not produced by human enzymes but only by bacterial metabolism in the colon from undigested protein containing p-[ring-^2^H_4_]tyrosine. It is readily absorbed from the colonic lumen and excreted in urine after sulfate- or glucuronide conjugation in the mucosa and liver. As p-[ring-^2^H_4_]cresol does not accumulate in the body of healthy subjects, its urinary excretion reflects its colonic generation (23).

Protein digestion was lower after RYGB compared to CTR and SG, and correlated negatively with protein malabsorption. Most likely, the lower protein digestion after RYGB is also due to the reduced stimulation of the pancreas and lower absorption surface due to the anatomical rearrangements. Indeed, secretion rates of pancreatic trypsin were lower in serial aspirates from the common channel of 13 RYGB patients than in the duodenal aspirates of 7 healthy controls (29). Furthermore, the time to maximal protein digestion rate was shorter after RYGB and SG compared to controls and probably reflects a faster gastric emptying rate. We were not able to directly measure gastric emptying rate as the isotopes that are standardly used in gastric emptying breath tests (^13^C or ^14^C) were already used for protein digestion and bile acid malabsorption, respectively. Similarly, a recent study that applied an isotope dilution technique to compare the absorption of intrinsically ^15^N labeled caseinate in RYGB and SG patients to controls found a higher systemic appearance rate of ingested phenylalanine in RYGB patients, indicating a faster protein digestion and suggesting a faster gastric emptying. However, total (6h) systemic recovery of ingested phenylalanine was similar across the 3 groups, suggesting that protein assimilation was unaffected after RYGB (35). A potential explanation for the apparent discrepancy with the results in the current study might lie in the different type of test meal (solid vs. liquid) in both studies. We used a solid meal that requires mechanical degradation in the stomach to particles less than 1–2 mm in size before they can pass through the pyloric sphincter to the duodenum (36). As the gastric pouch of RYGB no longer has a pyloric sphincter to govern the passage of the stomach content to the small intestine, larger particles may enter the jejunum, contributing to the compromised intestinal digestion. This complication plays no part when using liquid test meals neither in SG patients, where the pyloric sphincter and the small intestinal anatomy remain conserved.

Protein fermentation is increased after RYGB but also after SG, despite a normal protein digestion and absorption in the latter group, suggesting that other factors than protein malabsorption are involved. In addition to the type and amount of substrates supplied to the colon and the composition of the microbiota, transit time is a major factor that determines microbial metabolism (37). In a cross-sectional study in 98 adults with increased metabolic risk, a slow colonic transit was associated with a shift from carbohydrate fermentation to protein fermentation (38). In the present study, total transit was slower in both surgery groups compared to controls, and was negatively associated with protein fermentation. Constipation is a common problem after BS (39) that has been, at least partly, explained by a low fiber intake. Nevertheless, fiber intake and transit time were not correlated in our study. An alternative explanation for the slow transit might be the reported increase in post-prandial levels of glucagon-like peptide-1 (GLP-1) and peptide-YY (PYY) after bariatric surgery which delay intestinal transit (40). Unfortunately, these gut hormones were not quantified in this study.

The modest increase in breath hydrogen in RYGB patients indicates that the carbohydrate fraction in the test meal (11.6 g of starch, 12 g of sucrose, and 1.5 g of lactose) is incompletely digested in some patients. Carbohydrate digestion requires pancreatic α-amylase to hydrolyze starch into maltose, maltotriose and α-limit dextrins and the brushborder disaccharidases sucrase-isomaltase, lactase, maltase-glucoamylase and trehalase that further convert disaccharides into monosaccharides. The amount of lactose in the test meal is probably too low to explain the increased hydrogen since even lactase-deficient subjects are able to digest up to 12 g lactose per day (41). Studies that administered glucose and measured its systemic absorption found no effect of RYGB (35, 42), suggesting that carbohydrate digestion rather than absorption is reduced. Since pancreatic amylase is a very stable enzyme that is secreted in a large excess, the impaired carbohydrate absorption is probably not due to a lack of pancreatic enzyme but either to asynchronization between the secretion and nutrient passage or a too short contact time with the brush border enzymes (30). Nevertheless, supplementation with oral pancreatic enzymes in RYGB patients suppressed the late rise in breath H_2_ (28).

The implications of the macronutrient malabsorption after RYGB on the nutritional status of the patient may be limited. The energy deficit due to malabsorption has been estimated at not more than 200 kcal/day which is considerably lower than the energy deficit induced by the lower food intake (42). However, the increase in protein fermentation observed in both surgery groups may require further investigation. Indeed, despite prospective cohort studies consistently reporting a substantial reduction in all-cancer risk and mortality, observational data suggest that colorectal cancer (CRC) risk might actually increase after BS (43). A plausible hypothesis, raised by Hull et al. (43), implies that changes in colonic microbial metabolites due to altered dietary intake, macronutrient malabsorption, altered transit and persistent low microbial diversity may drive colorectal carcinogenesis together with other pro-carcinogenic factors like exposure to secondary bile acids and local inflammation. However, this hypothesis needs to be further investigated.

The main strength of the study is the use of proteins intrinsically labeled with 3 different stable isotopes allowed estimating protein digestion, malabsorption and fermentation simultaneously and accurately. Unfortunately, such tests are not available for fat malabsorption, and therefore we estimated fat intake from dietary records and used the golden standard to measure fat excretion. Hydrogen excretion in breath is standardly measured as a qualitative indication of carbohydrate malabsorption.

A limitation of the study is the limited number of participants, although that was based on a sample size calculation using protein malabsorption as the outcome parameter. Unfortunately, we were able to only include 8 patients that underwent SG instead of 10. Moreover, the breath samples from one patient in the SG group were not of sufficient quality for reliable analysis resulting in a missing value for protein digestion. For 2 other parameters, one subject (not the same) was an outlier. Due to the small groups and to be transparent, we report the statistical analysis with and without the outlier included. The limited number of participants precluded more detailed investigation in the origin of the variability observed for several parameters, in particular in the RYGB groups. Several factors may have affected this variability such as the time after surgery, the amount of weight loss, the length of the biliopancreatic limb or dietary intake and should be further investigated in studies specifically designed for this purpose.

Finally, we eliminated subjects on lactose-, glutenfree, vegetarian and vegan diets which implicates that the results of the study can not be extrapolated to those groups. Animal protein is on average better digestible than plant based protein (44). Therefore, we expect that BS patients on a vegetarian or vegan protein might experience a higher rather than lower degree of protein malabsorption.

In conclusion, RYGB but not SG patients experience higher fat, carbohydrate and protein malabsorption compared to controls. Bile acid malabsorption was not a driver for the fat malabsorption. Protein fermentation was increased after both surgery types and was associated with a slow transit. It would be interesting to evaluate whether treating the post-operative constipation would normalize the protein fermentation. Furthermore, the implications of the increased protein fermentation on colonic health need further investigation.

## Data availability statement

The raw data supporting the conclusions of this article will be made available by the authors, without undue reservation.

## Ethics statement

The studies involving human participants were reviewed and approved by the Ethics Committee of UZ/KU Leuven. The patients/participants provided their written informed consent to participate in this study.

## Author contributions

CE and KV acquired funding, conceived and designed the study, wrote the manuscript, and had primary responsibility for the final content. DV and JB provided essential materials. CE, AL, and GV conducted the research. CE performed the statistical analyses. ML, BV, and KV supervised the study. All authors read and approved the final manuscript.
